# A Co-Doping Materials Design Strategy for Selective Ozone Electrocatalysts

**DOI:** 10.1021/acs.jpclett.4c01150

**Published:** 2024-07-11

**Authors:** Rayan Alaufey, John A. Keith, Maureen Tang

**Affiliations:** † Department of Chemical and Biological Engineering, Drexel University, 3141 Chestnut Street, Philadelphia, Pennsylvania 19104, United States; ‡ Department of Chemical and Petroleum Engineering, University of Pittsburgh, 3700 O’Hara Street, Pittsburgh, Pennsylvania 15261, United States

## Abstract

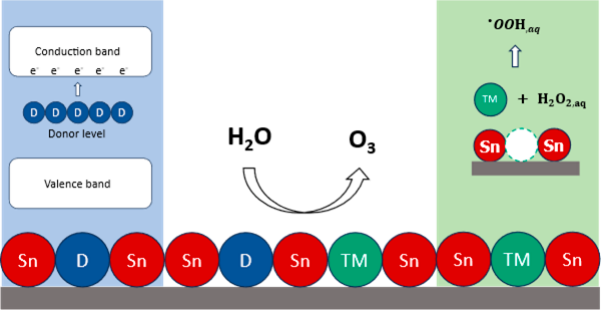

Catalysts for electrochemical ozone production (EOP) face inherent selectivity challenges stemming from thermodynamic constraints. This work establishes a design strategy for minimizing these limitations and inducing EOP activity in tin oxide, which is an intrinsically EOP-inactive material. We propose that selective ozone production using tin oxide catalysts can be broadly achieved by co-doping with two elements: first, n-type dopants to enhance electrical conductivity, and second, transition metal dopants that leach and homogeneously generate essential hydroperoxyl radical intermediates. Synthesizing tantalum, antimony, and tungsten n-type dopants with nickel, cobalt, and iron as transition metal dopants confirms that properly co-doping tin oxide yields EOP-active catalysts. This study offers a robust framework for advancing EOP catalyst design and serves as a case study for the application of fundamental co-catalysis and solid-state physics principles to induce catalytic activity in inert materials.

Advancements in electrochemical water treatment offer promising avenues for addressing global water scarcity.^1^ Among these methods, six-electron electrochemical ozone production (EOP) holds significant potential. Ozone (O_3_) is a potent oxidizer with a lower environmental impact than traditional disinfectants, and it can be generated on site through EOP.^2−5^ Furthermore, EOP produces hydrogen as a byproduct, potentially enhancing the cost-effectiveness of water treatment.^6^
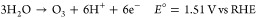
1
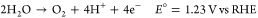
2

Despite its promise, EOP catalysts are hindered by low selectivity due to the thermodynamic favorability of the competing oxygen evolution reaction (OER).^7,8^ Therefore, the most promising catalysts for EOP possess low OER activity.^9,10^ Among these catalysts, nickel- and antimony-doped tin oxide (Ni/Sb-SnO_2_) stands out due to its low toxicity and superior selectivity. Importantly, SnO_2_ and Sb-SnO_2_ do not produce O_3_. Only Ni/Sb-SnO_2_ has been reported to be EOP-active under normal conditions.^11−13^

We previously investigated the mechanism of EOP on Ni/Sb-SnO_2_.^14^ We postulated that electrochemical oxidations on SnO_2_ is governed by transient hydrogen peroxide (H_2_O_2_) generation, which is produced via two-electron water oxidation. We further proposed that H_2_O_2_ is catalyzed by leached Ni^4+^ cations to solution-phase hydroperoxyl radicals (^•^OOH) by a homogeneous pseudo-Fenton reaction. These radicals are subsequently electrochemically oxidized, which leads to O_3_ generation. Despite not being able to directly detect it, invoking a transient H_2_O_2_ intermediate explained all of the experimental observations in our system, and the feasibility of this mechanism was supported by spectroscopic detection of reactive oxygen species, electroanalysis, and quantum chemistry calculations:^14^
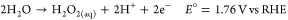
3
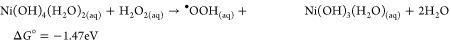
4
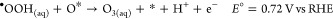
5Furthermore, we found that Sb functions as an n-type dopant that increases electrical conductivity, thereby enabling higher electrochemical activity, which is consistent with findings on transparent conductive oxides and OER catalyst supports.^15−19^

These previous results suggest a broader co-doping design strategy in which any combination of an n-type dopant that increases electrical conductivity and a transition metal (TM) dopant capable of homogeneously generating ^•^OOH can induce EOP activity in SnO_2_. This hypothesis challenges previous claims, including our own, regarding Ni’s unique role in inducing EOP activity.^3,4,8,10,11^ In this work, we validate this hypothesis by synthesizing co-doped SnO_2_ catalysts with tantalum (Ta), antimony (Sb), and tungsten (W) n-type dopants combined with nickel (Ni), cobalt (Co), and iron (Fe) as TM dopants. The proposed co-doping strategy builds on established solid-state doping principles and draws inspiration from existing co-catalysis approaches, which have proven to be successful in tailoring material properties for bimetallic CO_2_ reduction and OER, among other reactions.^20−24^

We found that synthetic parameters are responsible for the apparently unique effects of Ni. Wang et al. first reported the activity of Ni/Sb-SnO_2_ towards EOP along with observations that alternative TMs, including Co and Fe, did not exhibit O_3_ activity.^10,11^ To the best of our knowledge, there have been no additional reports on the lack of EOP activity using alternative TMs. To understand why attempts to induce EOP activity via Co and Fe doping using the simple sol–gel approach employed by Wang et al. have been unsuccessful, we analyzed the surface composition of the electrodes after drying but before calcination. The X-ray photoelectron spectroscopy (XPS) results depicted in Figure 1 revealed that Ni was present on the film surface, while Co was undetectable despite being initially added in equimolar amounts, suggesting that more Ni is incorporated into the catalyst.

**Figure 1:**
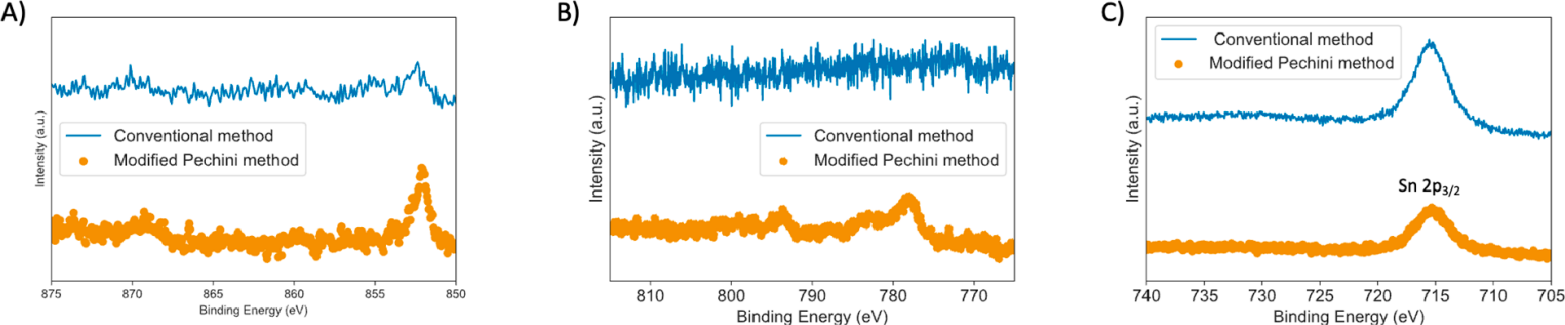
Transition metal region XPS spectra for electrodes after drying using the conventional sol–gel method and the modified Pechini method for the (A) Ni 2p region, (B) Co 2p region, and (C) Fe 2p region (overlaps with the Sn 2p_3/2_ orbital).

This trend might be attributed to segregation and evaporation processes due to hydrolysis and condensation kinetics that differ by orders of magnitude for different transition metal precursors.^25^ The presence of Fe on the film surface could not be confirmed by XPS, as its 2p orbital overlaps with the Sn 2p_3/2_ orbital as shown in Figure 1C, with weaker Fe peaks undetectable at this low doping concentration (1 mol % in the precursor solution).^26^ We note that at the employed concentration of the TMs they are not detectable on the electrode surface after calcining as shown in Figure S6.

To ensure the incorporation of more Fe and Co into the catalyst, we adopted a modified Pechini method utilizing anhydrous precursors and chelating agents. The complete details of the synthesis are provided in the Supporting Information. XPS spectra in panels A and B of Figure 1 revealed the presence of Co and Ni on the film surface after drying when this method was employed. Furthermore, comparison of the cyclic voltammograms for Fe-SnO_2_ made using the two methods depicted in Figure S2 shows that Fe oxidation features, discussed below, are present only when the modified Pechini method was employed, indicating the incorporation of more Fe into the SnO_2_ host with this synthetic route. The X-ray diffraction (XRD) patterns for all catalysts synthesized by the modified Pechini method in Figure S3 exhibit the characteristic rutile crystal structure of SnO_2_. The XPS spectra of Sn, n-type dopants, and TM dopants are shown in Figures S4–S6, respectively.

Voltammetry and spectroscopic detection of radicals suggest that leached TMs catalyze H_2_O_2_ to form solution-phase ^•^OOH, which ultimately leads to O_3_ production (eq-5).^14^ Two distinct peaks appear in the initial cyclic voltammograms in Figure 2A–C. We tentatively attribute the peak near 1.3 V to the initial oxidation of TM^2+^ to TM^3+^ and the second peak near 2.3 V to the oxidation of TM^3+^ to TM^4+^. These peaks are not present in the control cyclic voltammograms without TM dopants, shown in Figure S7, and suggest that TMs are present in multiple oxidation states under the reaction conditions. Although the redox potentials of TM^2+^/TM^3+^ and TM^3+^/TM^4+^ in 0.5 M H_2_SO_4_ are respectively lower than1.3 and 2.3 V according to the Pourbaix diagrams for Ni, Co, and Fe, incorporation into the SnO_2_ host at low concentrations can significantly increase the oxidation potentials.^13,27−29^ These peaks are absent in subsequent scans (shown in Figure S8), demonstrating that TMs leach during electrolysis. Control cyclic voltammograms with only TM dopants (no n-type dopants) are also shown in Figure S9. The leached TM cations can facilitate homogeneous pseudo-Fenton reactions with transient H_2_O_2_, leading to the generation of solution-phase ^•^OOH. The production of ^•^OOH on all nine catalysts is further evidenced by the absorbance spectra of 2-hydroxyethidium, the selective product of ^•^OOH and dihydroethidium, in Figure 2D.^30−32^ We note that in the absence of TM dopants, n-type singly doped SnO_2_ catalysts in Figure S10 did not generate any detectable 2-hydroxyethidium. Combined, Figure 2 demonstrates the homogeneous role of leached TM dopants in catalyzing EOP on SnO_2_-based catalysts.

**Figure 2:**
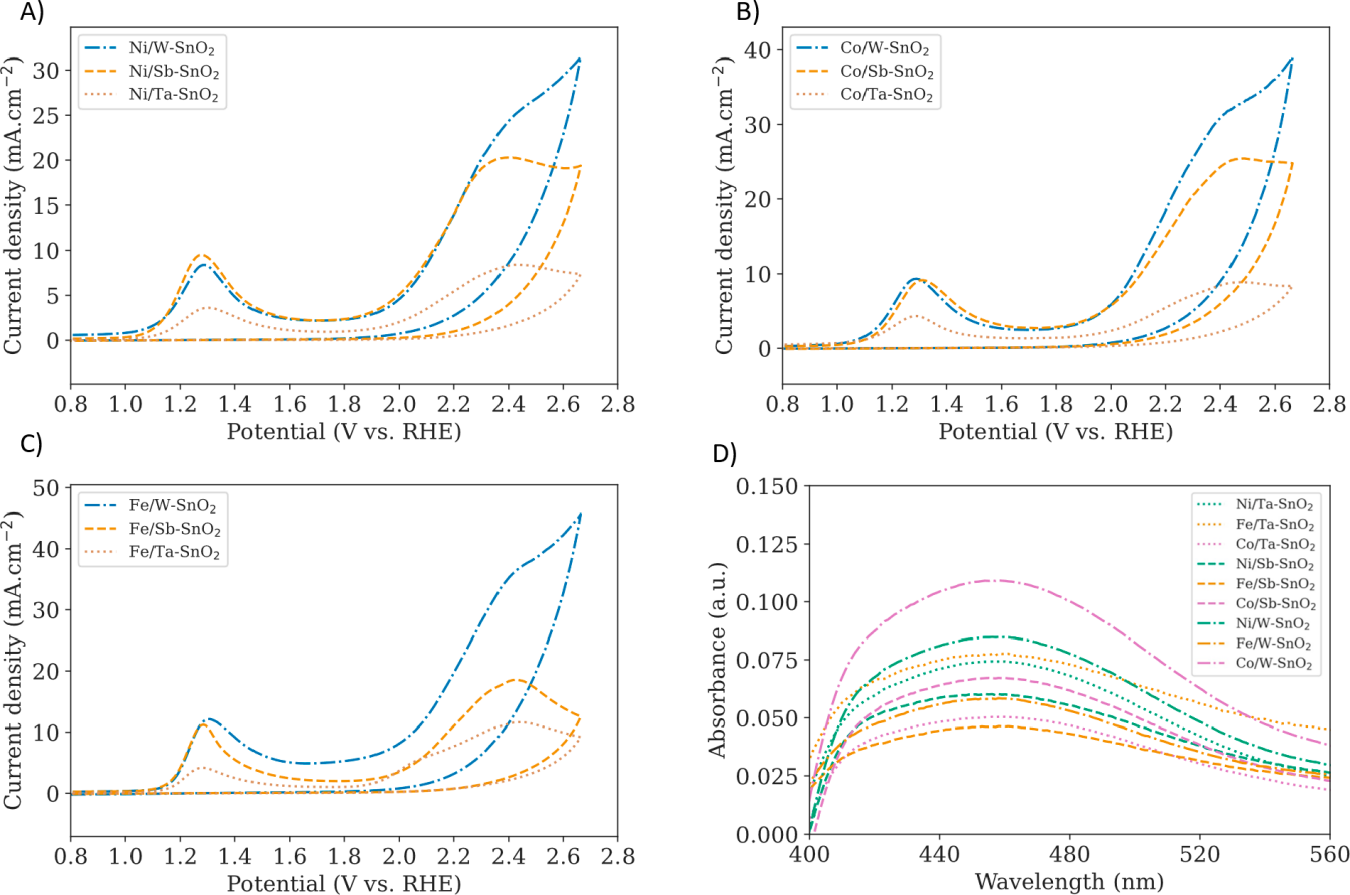
(A) Cyclic voltammograms in 0.5 M H_2_SO_4_ taken with a scan rate of 75 mV s^–1^ for (A) Ni-doped catalysts, (B) Co-doped catalysts, and (C) Fe-doped catalysts. (D) Absorbance spectra of 2-hydroxyethidium (^•^OOH probe) for all catalysts.

While the main role of leached TMs in EOP is generating ^•^OOH, they also have varying activities for side reactions with EOP intermediates and the O_3_ itself. Under the employed synthesis conditions, Ni and Co exhibited high selectivity for EOP whereas Fe did not. This aligns with the established role of Fe cations in converting residual O_3_ to O_2_ and H_2_O_2_ into hydroxyl radicals (^•^OH), which are not EOP intermediates.^14,33−36^ The high activity of Fe-doped catalysts for O_2_ hindered quantitative analysis due to the continuous stripping and decomposition of O_3_ by the generated O_2_. However, a starch test yielded qualitative evidence of EOP activity, as shown in Figure S11. Interestingly, decreasing the concentration of Fe in the precursor 100 times resulted in a moderate selectivity increase toward EOP as demonstrated by “dilute” Fe/Ta-SnO_2_ in Figure S12. Because Ni and Co displayed similar activities under identical conditions, further quantitative analysis focuses on these two dopants.

In contrast to TM dopants, we propose that n-type dopants are catalytically inert. However, they are essential for enhancing electrochemical activity. Pure SnO_2_ is a wide bandgap semiconductor with low electrical conductivity.^37,38^ The properties of pure SnO_2_ prepared using the modified Pechini method outlined in this study are shown in Figure S13. Typically, donor levels are shallow for a reducible and non-oxidizable oxide, such as SnO_2_. In contrast, acceptor levels are deep, rendering it amenable to n-type but not p-type doping.^39−41^ For n-type doping, a donor atom with one more electron than the host is picked to increase its conductivity. Therefore, catalyst conductivity can be increased by substituting Sn^4+^ with 5+ donor cations.^17,18,41,43^

Figure 3A demonstrates that all n-type dopants successfully enhanced electrical conductivity, which is attributed to effective dopant integration within the host structure and their presence, at least partially, in the 5+ oxidation state, as discussed in Figures S3 and S5. W-doped catalysts displayed the highest conductivity, followed by catalysts doped with Sb and Ta. On the basis of the reported variation of conductivity values in the literature, we attribute this trend to the synthesis conditions rather than to the intrinsic dopant properties.^17,18,43,44^ Thus, W may not always be better than Sb and Ta at increasing SnO_2_ conductivity across all synthesis conditions and precursor ratios. As expected, enhanced conductivity correlates with lower charge transfer resistance under the reaction conditions (2.70 V vs RHE, 0.50 M H_2_SO_4_), as shown in the Nyquist plots in Figure 3B, and with total current densities, as shown in Figure 2A–C. Electrochemical analysis and microscopy show that the surface area is not a major factor in performance variations. Table S14 and SEM micrographs in Figure S15 reveal no notable differences in double-layer capacitance values or surface morphology, suggesting comparable active surface areas across all electrodes.

**Figure 3:**
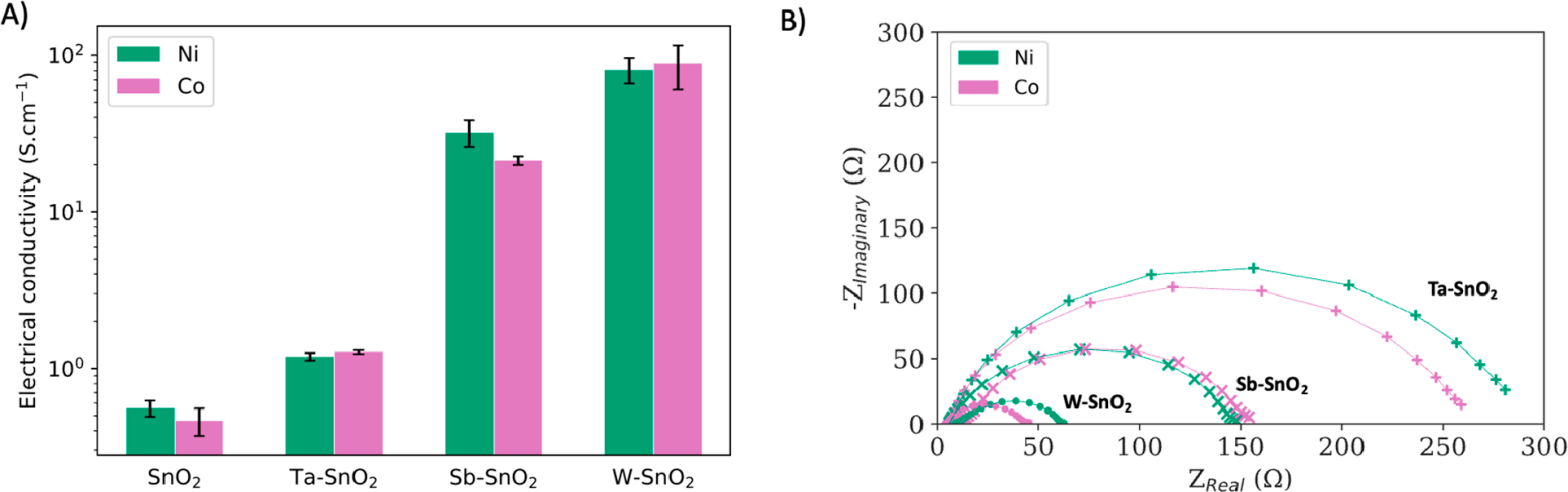
(A) Electrical conductivity of catalysts with different n-type dopants. Three samples are shown. (B) Nyquist plots obtained using EIS under the reaction conditions (0.5 M H_2_SO_4_, 2.70 V vs RHE) demonstrating that higher electrical conductivity correlates with lower charge transfer resistance.

Panels A and B of Figure 4 show the molar fluxes and current efficiencies, respectively, for EOP on Ni- and Co-doped catalysts. Values for Sb-doped catalysts are consistent with the literature.^4,6,12,33^ The effect of Ta and W doping yields a maximum conductivity for EOP activity and selectivity. We note that similar to singly doped Sb-SnO_2_ (no TM dopants), singly doped Ta-SnO_2_, and W-SnO_2_ did not generate detectable O_3_. The compositions tested here were not optimized for performance and do not prove any specific dopant combination is necessarily superior for EOP across all experimental conditions. One explanation for the observation in Figure 4 is the competition between EOP and the two-electron electrochemical oxidation of H_2_O_2_ to O_2_:
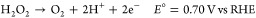
6As proposed in previous work, once transient H_2_O_2_ forms, it can either undergo direct electro-oxidation on the electrode surface or react homogeneously to form solution-phase ^•^OOH.^14^ While higher conductivity has no effect on the homogeneous pseudo-Fenton step, it intuitively enhances the charge transfer kinetics of all surface reactions, as supported by Figure 3B. Therefore, increased conductivity simultaneously promotes both H_2_O_2_ generation and its heterogeneous consumption to form O_2_, increasing the total current but reducing EOP activity and selectivity.

**Figure 4:**
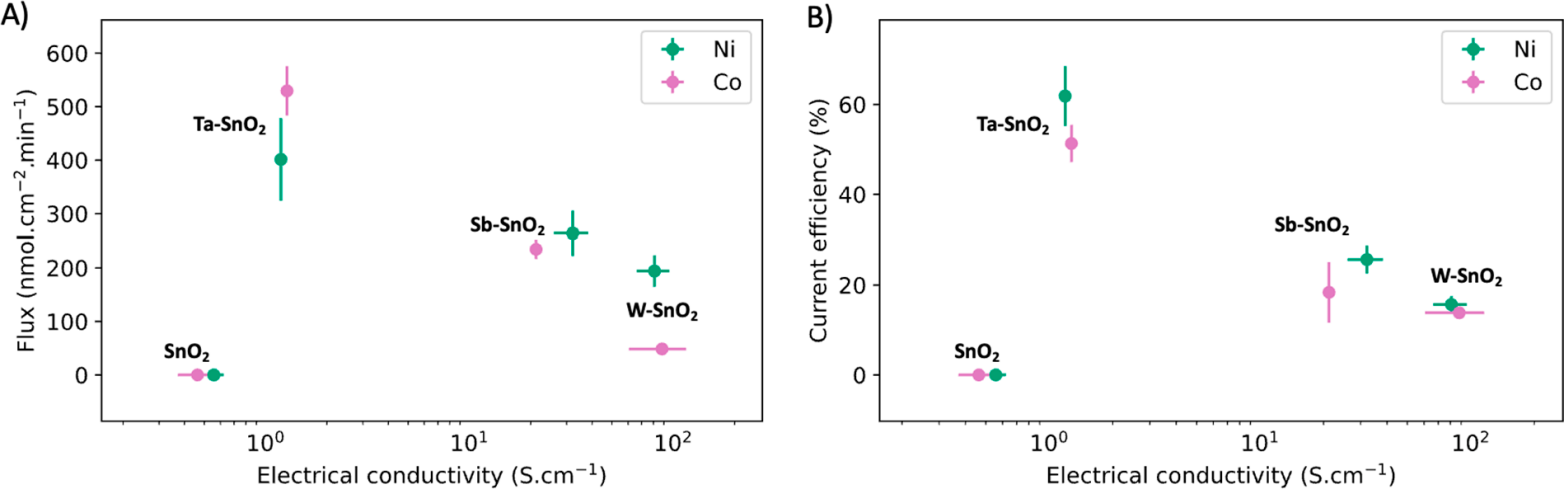
Relationship between the electrical conductivity and EOP performance: (A) O_3_ flux vs conductivity and (B) current efficiency vs conductivity. O_3_ was generated at 2.70 V vs RHE in 0.5 M H_2_SO_4_. Electrical conductivity was measured using a four-point probe. Dots represent the average of three samples, and error bars represent the standard error.

Overall, our results reveal two key requirements for inducing EOP activity in SnO_2_. First, the catalyst’s conductivity must be enhanced, which can be achieved through n-type doping. However, excessively high electrical conductivity impedes the production of O_3_ likely by improving the kinetics of electrochemical H_2_O_2_ decomposition. Second, ^.•^OOH generation is crucial. This can be facilitated by doping with TMs capable of promoting homogeneous radical formation. On the basis of these guidelines, we anticipate that strategically combining dopants for optimized conductivity and ^•^OOH production will lead to highly active and selective EOP catalysts.

More work is required to design and discover catalysts suitable for commercial devices. Notably, stability in acid, a major limitation to SnO_2_-based catalysts, remains a major limitation for the practical application of these catalysts, as shown in Figure S16.^15,19,46,47^ Future work should explore if this co-doping strategy can be used to induce EOP activity in inert oxides beyond SnO_2_. Additionally, homogeneous generation of solution-phase intermediates through TM doping presents exciting possibilities for co-catalyzing novel multistep electrocatalytic processes.

## Supporting Information Available

Experimental methods, XRD patterns, lattice parameters, XPS spectra, control electrochemical cyclic voltammetry, flux and selectivity for diluted Fe/Ta-SnO_2_, double-layer capacitance values, and scanning electron microscopy micrographs (PDF)
Transparent Peer Review report available (PDF)
