# Supplementary material for: A Co-Doping Materials Design Strategy for Selective Ozone Electrocatalysts
Source: J Phys Chem Lett. 2024 Jul 11;15(28):7351–6. doi: 10.1021/acs.jpclett.4c01150 (PMC11261613; doi:10.1021/acs.jpclett.4c01150)
Supplement: Supplementary file 1 — jz4c01150_si_001.pdf [file jz4c01150_si_001.pdf]

A Co-Doping Materials Design Strategy for Selective Ozone Electrocatalysts

Rayan Alaufey[a], John A. Keith[b], Maureen Tang*[a]

[a] Department of Chemical and Biological Engineering, Drexel University, 3141 Chestnut Street, Philadelphia PA, 19104

[b] Department of Chemical and Petroleum Engineering, University of Pittsburgh, 3700 O'Hara Street, Pittsburgh, PA 15261

* Corresponding author: mhtang@drexel.edu

Table of Contents

S1. Experimental methods **2**

A. Catalyst Synthesis **2**

B. Characterization **2**

C. Electrochemical testing and Ozone measurements **3**

D. Free radical detection **4**

S2. CVs for Fe-SnO_2 synthesized with conventional method and modified Pechini method **5**

S3. XRD patterns for catalysts made with the modified Pechini method. **6**

S4. Sn 3d XPS spectrum **7**

S5. N-type dopants XPS spectra **8**

S6. Transition metal dopants XPS spectra. **9**

S7. Control CVs with no transition metal dopants. **10**

S8. Subsequent CVs after transition metals leaching **11**

S9. Control CVs with no n-type dopants. **12**

S10. Control 2-hydroxy ethidium absorbance spectra for catalysts without TM dopants. **13**

S11. Qualitative confirmation of O_3 generation for Fe doped SnO_2. .... **14**

S12. O_3 molar flux and current efficiency for dilute Fe/Ta-SnO_2 .... **15**

S13. Characterization of un-doped SnO_2 synthesized with the modified Pechini method **16**

S14. Double layer capacitance and charge transfer resistance of Ni and Co doped catalysts .. **17**

S15. Representative SEM micrographs **18**

S16. 24-hour constant potential stability tests **19**

S1. Experimental methods

A. Catalyst Synthesis

Electrodes were prepared using a modified Pechini method using anhydrous chloride precursors: $SnCl_4$, $SbCl_3$, WCl_6, $TaCl_5$, $NiCl_2$, $CoCl_2$, and $FeCl_2$. A precursor mole ratio of 96:3:1 Sn:D:TM was used where D is a donor n-type dopant (Sb,Ta, or W) and TM is a transition metal (Ni, Co, or Fe). In a typical synthesis, 15 mL of 2-propanol was heated to 60 °C on a hotplate followed by the addition of 4 grams of citric acid. The solution was heated and stirred until complete dissolution of citric acid was achieved. The solution was moved to an air-free glovebox where 2.5 mL of anhydrous tin chloride was slowly added to it. (CAUTION! The reaction is highly exothermic and volatile vapors of $SnCl_4$ are toxic). The appropriate amounts of dopants were subsequently added to the mixture. The mixture was immediately transferred out of the glovebox to a hotplate and mixed with 5 mL of dry ethylene glycol to give the final precursor solution. The final solution was then held at 80 °C and stirred for 72 hours. Ti foil was cut into 0.5x0.5 cm^2 substrates. The substrates were then chemically etched by boiling in 50 mL oxalic acid for 30 minutes. Etched substrates were washed and sonicated with Millipore water and immediately preheated to 85°C on a silicon carrier wafer to be used for electrode synthesis. The precursor solution was evenly drop-casted on each substrate. The samples were held at 185 °C for three minutes to allow for drying and then they were sintered at 700°C for five minutes inside a muffle furnace. After removal from the furnace, the samples were cooled in air and turned to the opposite side. The procedure was repeated nine more times for a total of ten applications (5 applications on each side). On the final application, the samples remained in the muffle furnace at 700°C for 3 hours. Finally, a Ti wire (0.125 mm thick) was spot-welded to one side of the electrode.

B. Characterization

X-ray photoemission spectroscopy (XPS) was performed using a Versa Probe 5000 spectrometer (Physical Electronics Inc., USA) with Al Kα radiation (monochromatic) and a beam setting of 200 μm (diameter), 25 W power, and 15 kV accelerating voltage. CasaXPS software was used

for peak fitting, with the adventitious carbon C 1s peak at 284.8 eV serving as the charge correction reference.[1] X-ray diffraction (XRD) measurements were conducted on a Rigaku Miniflex diffractometer using Bragg-Brentano geometry and a Cu Kα filter (λ = 1.54056 Å). Catalyst conductivity was determined using the 4-probe method on an Ossila T2001A3 Hall effect measurement system under 1 mA at room temperature. Prior to this, the catalyst films were deposited on quartz substrates using the same preparation conditions mentioned earlier.

C. Electrochemical testing and Ozone measurements

The experimental setup was adapted from previous work.[2,3] The electrocatalysts served as working electrodes, while a platinum wire and a BASI Ag/AgCl reference electrode in 3.0 M KCl were used as counter and reference electrodes, respectively. Each test utilized a freshly prepared solution and new working electrode within a 4.5 mL airtight quartz cuvette connected to a BioLogic potentiostat. 0.5 M H_2SO_4 served as the electrolyte.

Cyclic voltammetry measurements were performed with a scan rate of 75 mV.s^{-1}. For ozone (O_3) quantification, a constant potential of 2.70 V vs. RHE was applied for 1 minute. A fresh electrolyte was used for each test. A PerkinElmer Lambda 35 UV-Vis spectrometer measured O_3 absorbance at 258 nm, with a background measurement taken before each test. The O_3 concentration was determined using Beer's law and a molar extinction coefficient of 3000 M^{-1} cm^{-1}.[4,5] The molar electrode flux was then calculated using the following equation:

$$Flux = \frac{c_{O_3} \cdot V}{t \cdot A} \tag{1}$$

Where c_{O_3} is the concentration of ozone, V is the volume of the cell (4.5 mL), *t* is the electrolysis time (1 minute), and *A* is the geometric area of the electrode (0.5 cm^{-2}). Current efficiency was calculated using this equation:

$$CE = \frac{c_{O_3} \cdot V \cdot F \cdot z}{q} \tag{2}$$

Where F is faraday's constant (96,485 C.mol^{-1}), z is the number of electrons in EOP (6), and q is the total charge generated during electrolysis (C).

Electrochemical impedance spectroscopy (EIS) data was acquired using a potentiostatic mode. A small AC voltage perturbation of 15 mV was superimposed on the applied potential. The frequency range scanned was 10 kHz to 70 Hz with 10 data points per decade.

D. Free radical detection

Dihydroethidium (DHE) was employed to detect the presence of hydroperoxyl radicals through their reaction, forming 2-hydroxy ethidium (2-OH^+). A 30 μM stock solution of DHE was prepared beforehand. During the 1-minute electrolysis, 20 μL of the DHE stock solution was directly added onto the working electrode at the 50-second mark. Following this, the absorbance of the resulting 2-hydroxy ethidium was measured near 440 nm using a PerkinElmer Lambda 35 UV-Vis spectrophotometer.

S2. CVs for Fe-SnO_2 synthesized with conventional method and modified Pechini method

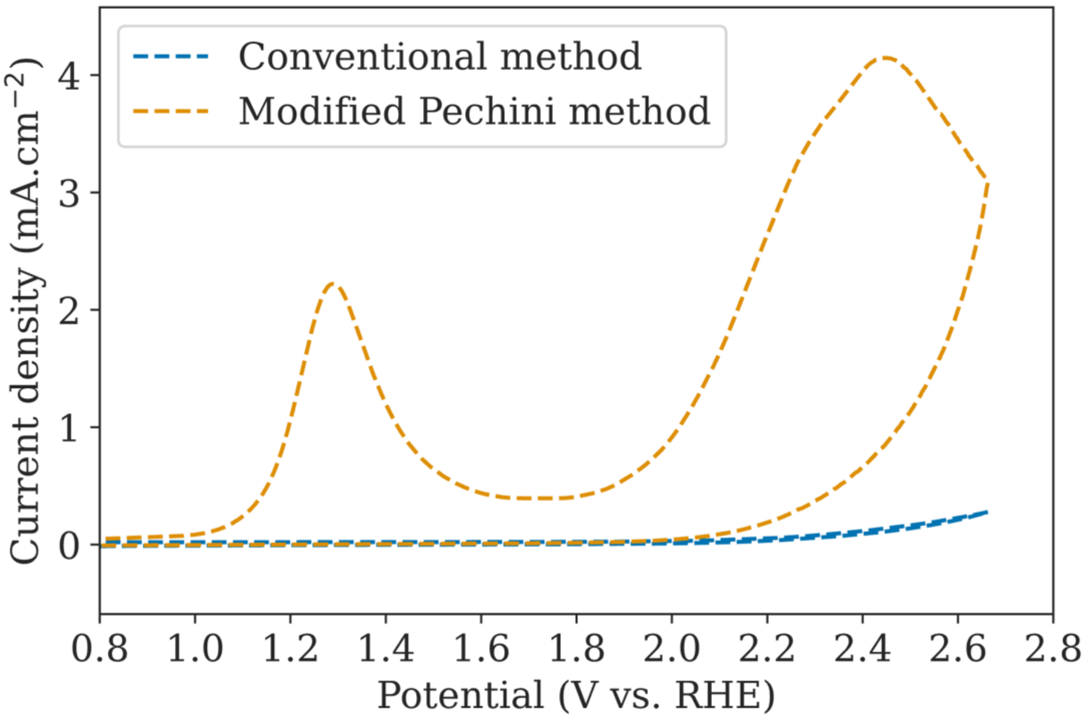

Figure S2: CVs for Fe-SnO_2 (no n-type dopants) in 0.5 M H_2SO_4 taken with a scan rate of 75 mV.s^{-1}

The current density generated by the electrode made with modified Pechini method was more than an order of magnitude greater than the electrode made with the conventional sol-gel method. We attribute this enhancement to the increased incorporation of Fe into the catalyst achieved through the modified Pechini method.

S3. XRD patterns for catalysts made with the modified Pechini method.

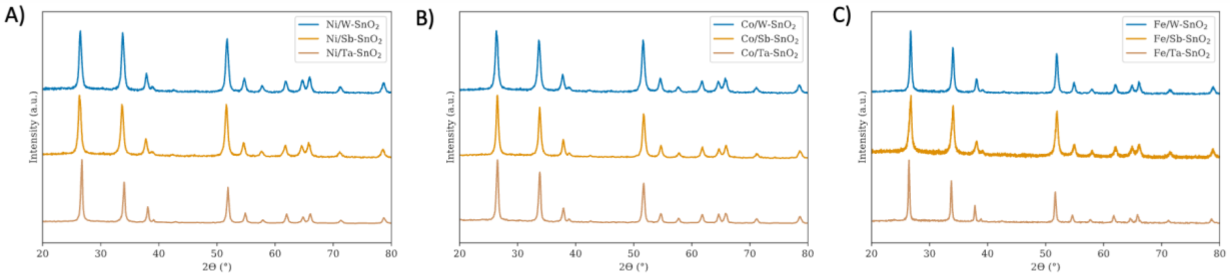

Figure S3: XRD patterns obtained for catalysts synthesized using the modified Pechini method, confirming the desired rutile crystal structure. A) Ni doped catalysts. B) Co doped catalyst. C) Fe doped catalysts.

Catalyst	a (A)	c (A)	Average crystallite size (nm)
Ni-Ta/SnO_2	4.75	3.18	24.2
Ni-Sb/SnO_2	4.74	3.19	20.5
Ni-W/SnO_2	4.74	3.19	20.5
Co-Ta/SnO_2	4.74	3.19	24.2
Co-Sb/SnO_2	4.74	3.19	22.3
Co-W/SnO_2	4.76	3.18	20.5
Fe-Ta/SnO_2	4.75	3.19	25.2
Fe-Sb/SnO_2	4.72	3.18	22.6
Fe-W/SnO_2	4.72	3.17	22.7

Table S3: lattice parameters and average crystalline size

Table S3 presents lattice parameters and average crystallite sizes for all catalysts. Catalysts doped with Fe were larger than those doped with Ni and Co. Furthermore, Ta-doped catalysts exhibit larger average crystallite sizes compared to those doped with Sb and W, which have similar sizes. There were no observed separate crystalline phases that can be attributed to any of the dopants.

S4. Sn 3d XPS spectrum

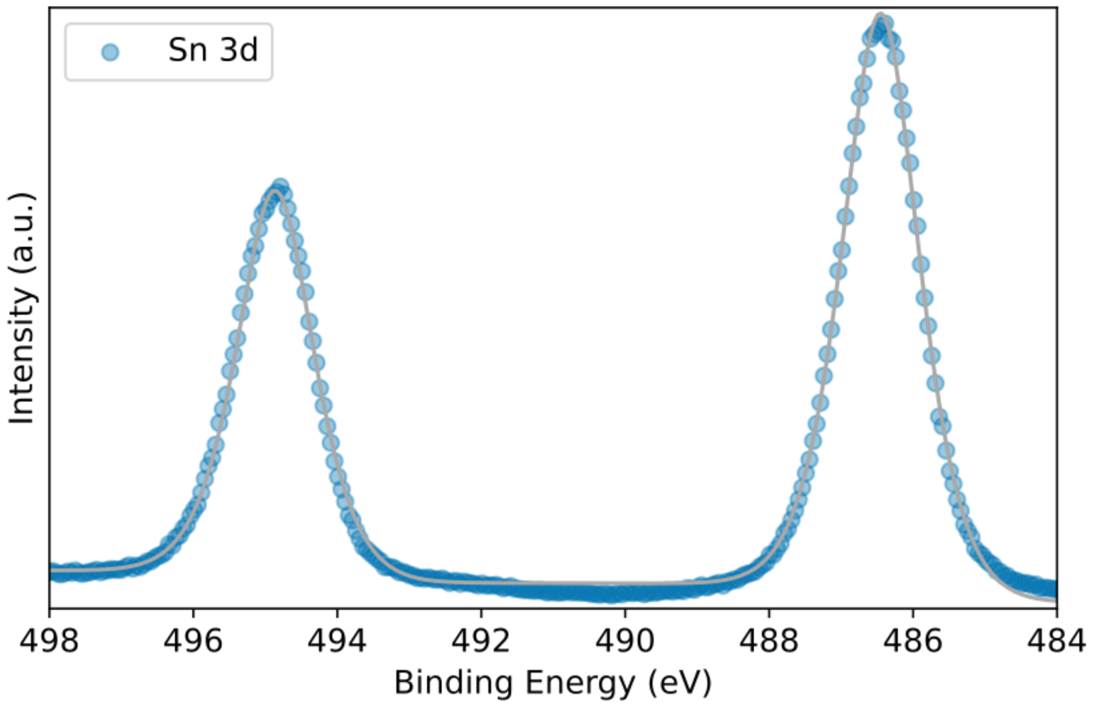

Figure S4: Sn 3d XPS spectrum.

The XPS spectrum in Figure S4 shows that Sn is exists predominantly present as Sn^{4+} with a minor amount of Sn^{2+}, although quantifying is challenging due to the overlap of the two peaks.[6]

S5. N-type dopants XPS spectra

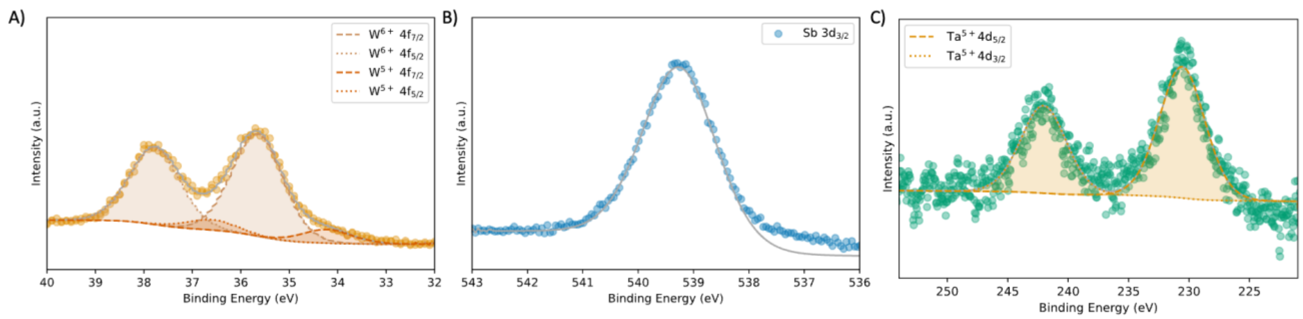

Figure S5: XPS spectra for the A) W 4f region B) Sb $3d_{3/2}$ region C) Ta 4d region.

The 4f tungsten spectrum in Figure S5 A shows that it was primarily present as W^{6+}, with a smaller amount present as W^{5+}.[9] The Sb $3d_{5/2}$ orbital overlaps with the O 1s peak. Therefore, the Sb $3d_{3/2}$ peak in Figure S5 B is used to monitor Sb. While the spectrum does not allow for quantification, Sb likely exists as a mix of Sb^{3+} and Sb^{5+}.[6,8] Ta is mainly present as Ta^{5+}, identified using the less common 4d region in Figure S5 C due to the overlap between the Sn 2s orbital and the more frequently analyzed 4f orbital. [7]

S6. Transition metal dopants XPS spectra.

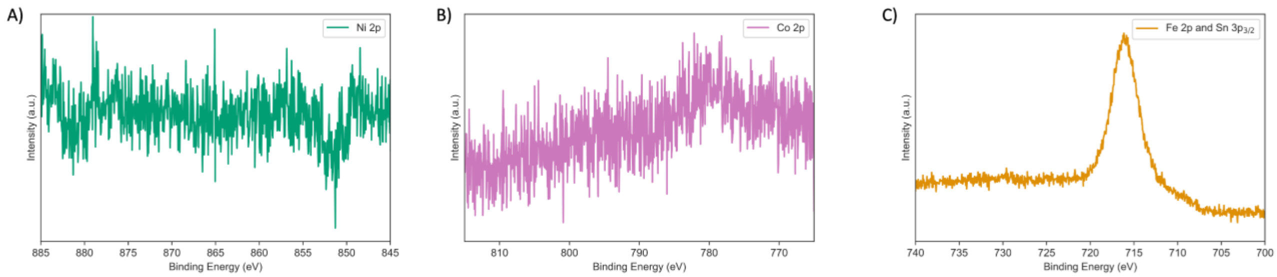

Figure S6: XPS spectra for the A) Ni 2p region B) Co 2p region C) Fe 2p region.

Ni and Co were not detected on the electrode surface as shown Figures S6 A and S6 B which aligns with literature at the used dopants ratio.[3,10–12] The Fe 2p orbital overlaps with the Sn $2p_{3/2}$ peak which complicates analysis as shown in Figure 6C, but we similarly expect it not to be present on the electrode surface.

S7. Control CVs with no transition metal dopants.

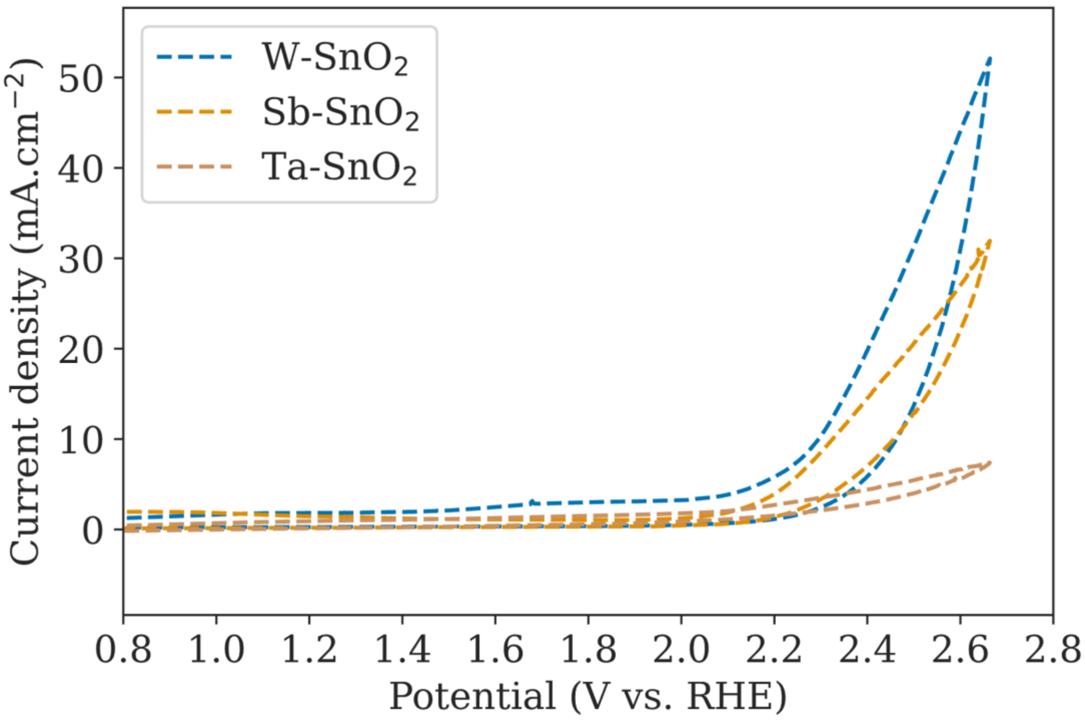

Figure S7: Control CVs for catalysts with no TM dopants in 0.5 M H_2SO_4 taken with a scan rate of 75 mV.s^{-1}

S8. Subsequent CVs after transition metals leaching

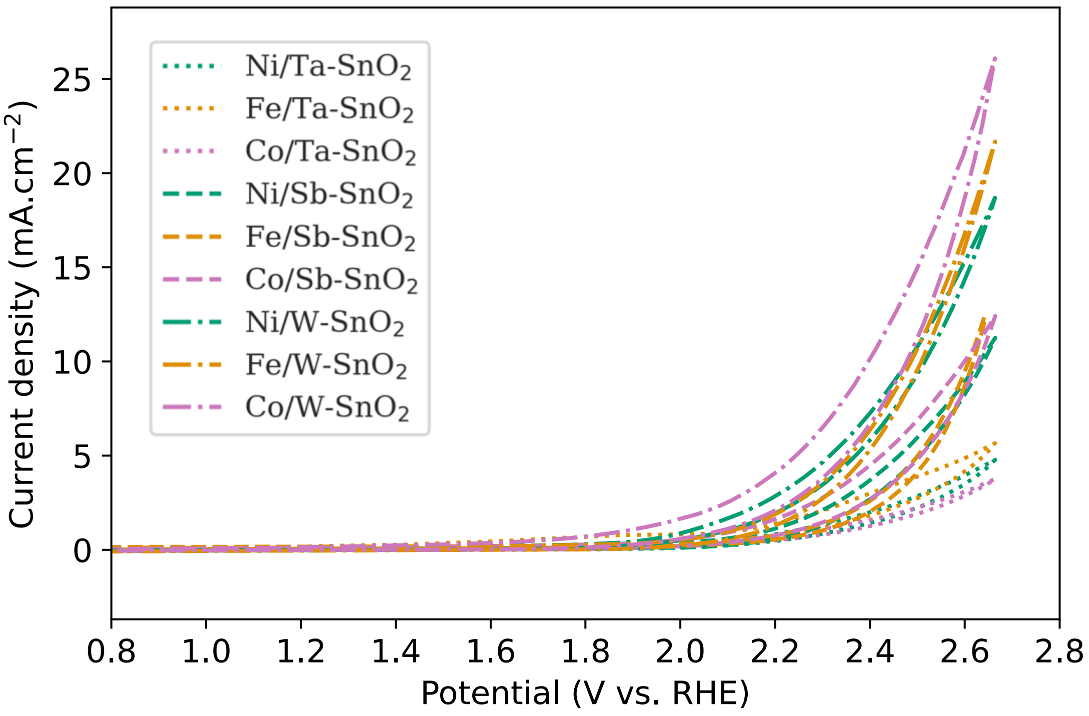

Figure S8: Subsequent CV cycles for different catalysts in 0.5 M H_2SO_4 taken with a scan rate of 75 mV.s^{-1}

S9. Control CVs with no n-type dopants.

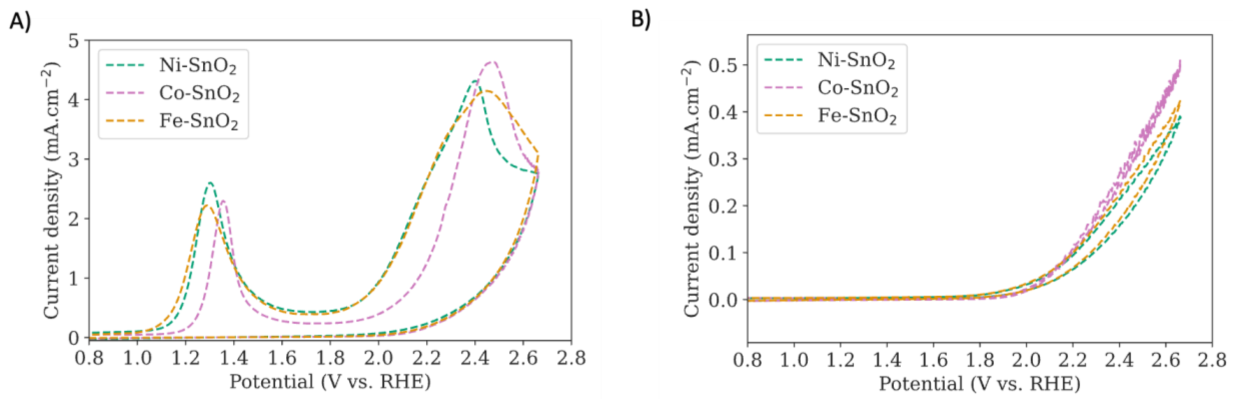

Figure S9: Control CVs for catalysts with no n-type dopants in 0.5 M H_2SO_4 taken with a scan rate of 75 $mV.s^{-1}$. A) 1st cycle. B) 3rd cycle.

The current density generated during the 1st scan cycle is more than an order of magnitude greater than current density generated during the 3rd scan cycle. Furthermore, the oxidation features observed in the 1st cycle disappear in the 3rd cycle. We attribute these observations to the oxidation and leaching of TM dopants during electrolysis in acid.

S10. Control 2-hydroxy ethidium absorbance spectra for catalysts without TM dopants.

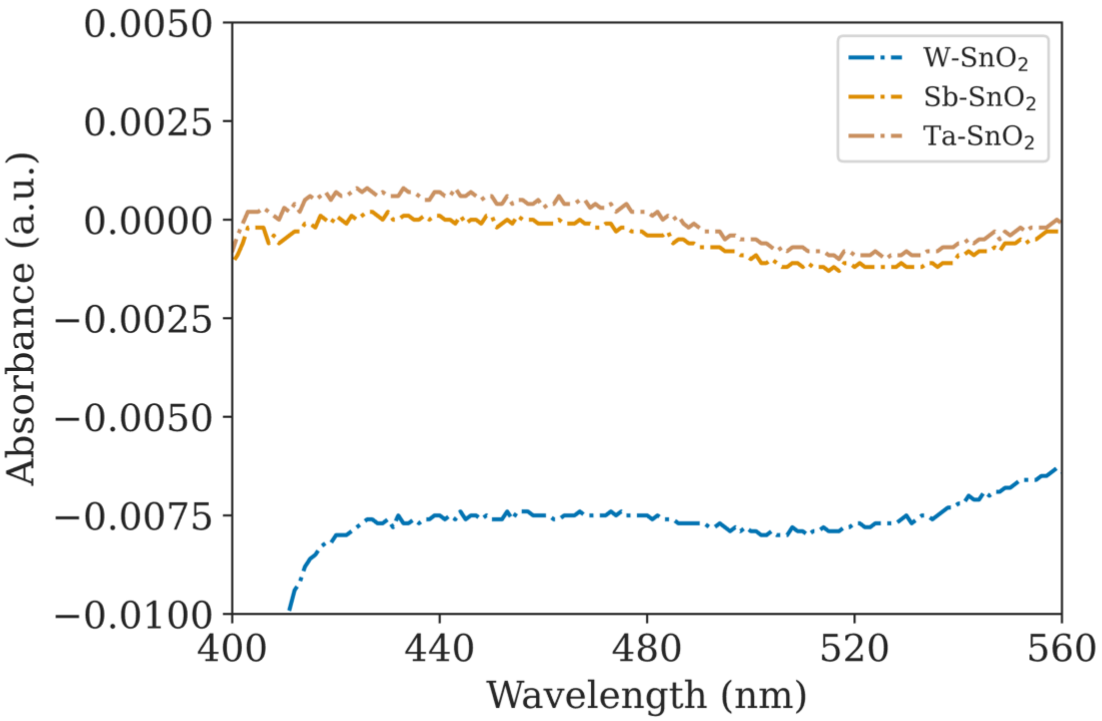

Figure S10: 2-hydroxy ethidium absorbance spectra for catalysts with no TM dopants (blank corrected)

S11. Qualitative confirmation of O_3 generation for Fe doped SnO_2.

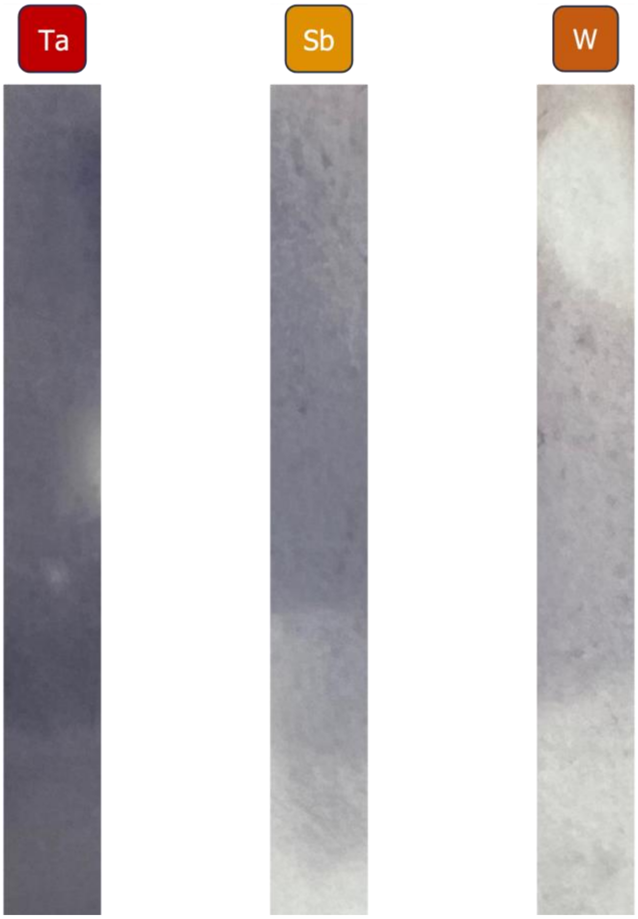

Figure S12: Starch paper indicator post 1-minute electrolysis with Fe doped catalysts.

In the presence of O_3, wet starch indicator turns from colorless to purple according to the following 2-step reaction:[13,14]

$$H_2O + 2KI + O_3 \rightarrow 2KOH + O_2 + I_2 \quad (3)$$

$$I_2 + Starch \rightarrow I_2 - Starch\ Complex\ (Purple) \quad (4)$$

S12. O_3 molar flux and current efficiency for dilute Fe/Ta-SnO_2

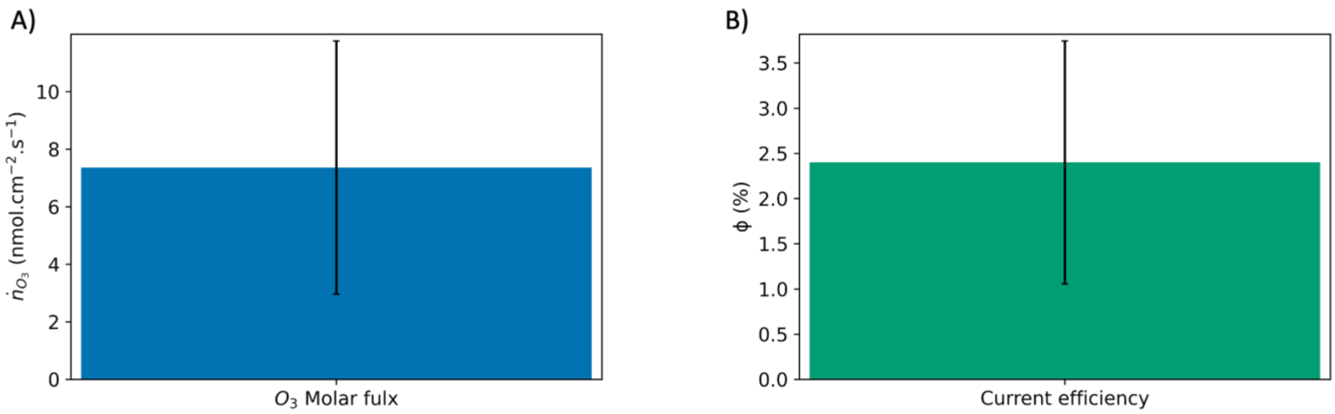

Figure S13: A) O_3 molar flux for dilute Fe/Ta-SnO_2 B) Current efficiency for dilute Fe/Ta-SnO_2

S13. Characterization of un-doped SnO_2 synthesized with the modified Pechini method

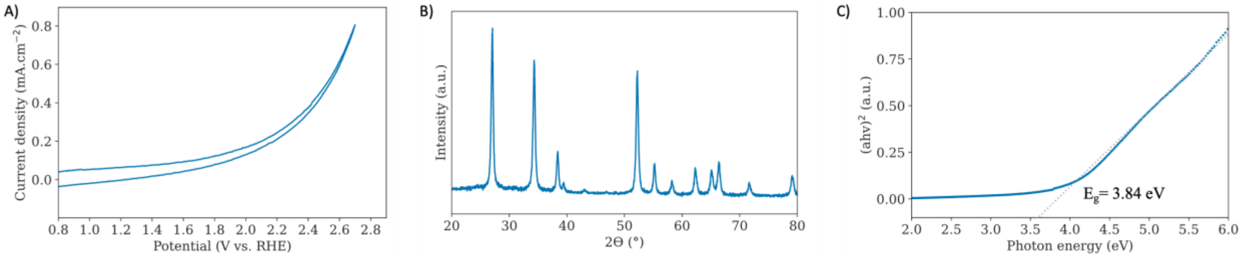

Figure S13: A) CV for undoped SnO_2 in 0.5 M H_2SO_4 taken with a scan rate of 75 mV.s^{-1}B) XRD data for undoped SnO_2 C) Tauc plot for undoped SnO_2

S14. Double layer capacitance and charge transfer resistance of Ni and Co doped catalysts

Catalyst	C_{DL} (μF.cm^{-1})	R_{CT} (Ω)
Ni-Ta/SnO_2	60 ± 11	273 ± 12
Ni-Sb/SnO_2	52 ± 6	132 ± 6
Ni-W/SnO_2	48 ± 7	49 ± 3
Co-Ta/SnO_2	52 ± 18	243 ± 10
Co-Sb/SnO_2	47 ± 14	139 ± 7
Co-W/SnO_2	52 ± 11	37 ± 2

Table S14: Double layer capacitance (C_{DL}) calculated from current scan rate dependence and charge transferer resistance (R_{CT}) under reaction conditions (2.70 V vs. RHE, 0.5 M H_2SO_4)

S15. Representative SEM micrographs

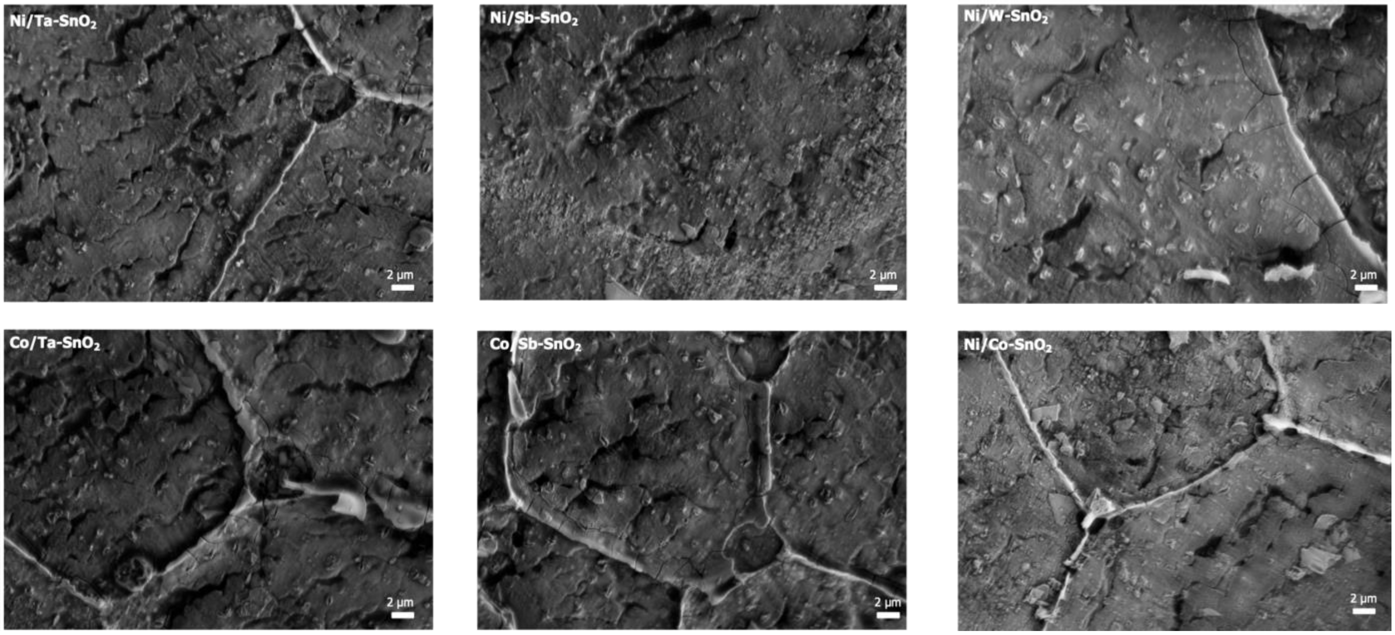

Figure S15: Representative SEM micrographs for catalysts with different n-type and TM dopants.

S16. 24-hour constant potential stability tests

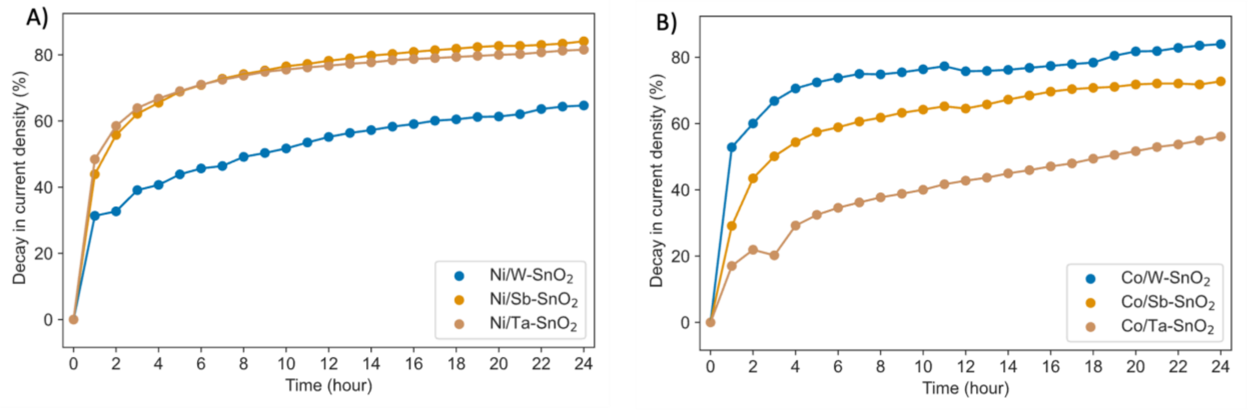

Figure S16: Decay in current density during potentiostatic (2.70 V vs RHE) electrolysis in 0.5 H_2SO_4

References

(1) Fairley, N.; Fernandez, V.; Richard-Plouet, M.; Guillot-Deudon, C.; Walton, J.; Smith, E.; Flahaut, D.; Greiner, M.; Biesinger, M.; Tougaard, S.; Morgan, D.; Baltrusaitis, J. Systematic and Collaborative Approach to Problem Solving Using X-Ray Photoelectron Spectroscopy. *Appl. Surf. Sci. Adv.* **2021**, *5*, 100112. https://doi.org/10.1016/j.apsadv.2021.100112.

(2) Wang, Y.-H.; Cheng, S.; Chan, K.-Y.; Li, X. Y. Electrolytic Generation of Ozone on Antimony- and Nickel-Doped Tin Oxide Electrode. *J. Electrochem. Soc.* **2005**, *152* (11), D197. https://doi.org/10.1149/1.2041007.

(3) Lees, C. M.; Lansing, J. L.; Morelly, S. L.; Lee, S. E.; Tang, M. H. Ni- and Sb-Doped SnO2 Electrocatalysts with High Current Efficiency for Ozone Production via Electrodeposited Nanostructures. *J. Electrochem. Soc.* **2018**, *165* (16), E833. https://doi.org/10.1149/2.0051816jes.

(4) Panich, N. M.; Ershov, B. G. Solubility and Stability of Ozone in Acetonitrile. *J. Mol. Liq.* **2021**, *340*, 117318. https://doi.org/10.1016/j.molliq.2021.117318.

(5) Buchan, K.; Martin-Robichaud, D.; Benfey, T. Measurement of Dissolved Ozone in Sea Water: A Comparison of Methods. *Aquac. Eng.* **2005**, *33*, 225–231. https://doi.org/10.1016/j.aquaeng.2005.02.002.

(6) Ponja, S. D.; Williamson, B. A. D.; Sathasivam, S.; Scanlon, D. O.; Parkin, I. P.; Carmalt, C. J. Enhanced Electrical Properties of Antimony Doped Tin Oxide Thin Films Deposited via Aerosol Assisted Chemical Vapour Deposition. *J. Mater. Chem. C* **2018**, *6* (27), 7257–7266. https://doi.org/10.1039/C8TC01929K.

(7) Jiménez-Morales, I.; Haidar, F.; Cavaliere, S.; Jones, D.; Rozière, J. Strong Interaction between Platinum Nanoparticles and Tantalum-Doped Tin Oxide Nanofibers and Its Activation and Stabilization Effects for Oxygen Reduction Reaction. *ACS Catal.* **2020**, *10* (18), 10399–10411. https://doi.org/10.1021/acscatal.0c02220.

(8) Williamson, B. A. D.; Featherstone, T. J.; Sathasivam, S. S.; Swallow, J. E. N.; Shiel, H.; Jones, L. A. H.; Smiles, M. J.; Regoutz, A.; Lee, T.-L.; Xia, X.; Blackman, C.; Thakur, P. K.; Carmalt, C. J.; Parkin, I. P.; Veal, T. D.; Scanlon, D. O. Resonant Ta Doping for Enhanced Mobility in Transparent Conducting SnO2. *Chem. Mater.* **2020**, *32* (5), 1964–1973. https://doi.org/10.1021/acs.chemmater.9b04845.

(9) Sathasivam, S.; Ponja, S. D.; Park, S.; Sanchez-Perez, C.; Blackman, C.; Parkin, I. P.; Carmalt, C. J. Highly Conductive Tungsten-Doped Tin(IV) Oxide Transparent Electrodes Delivered by Lattice-Strain Control. *ACS Appl. Energy Mater.* **2023**, *6* (11), 5835–5841. https://doi.org/10.1021/acsaem.3c00248.

(10) Christensen, P. A.; Yonar, T.; Zakaria, K. The Electrochemical Generation of Ozone: A Review. *Ozone Sci. Eng.* **2013**, *35* (3), 149–167. https://doi.org/10.1080/01919512.2013.761564.

(11) Malvankar, S.; Doke, S.; Gahlaut, R.; Martinez-Teran, E.; El-Gendy, A. A.; Deshpande, U.; Mahamuni, S. Co-Doped SnO2 Nanocrystals: XPS, Raman, and Magnetic Studies. *J. Electron. Mater.* **2020**, *49* (3), 1872–1880. https://doi.org/10.1007/s11664-019-07865-5.

(12) Toloman, D.; Popa, A.; Stefan, M.; Silipas, T. D.; Suciu, R. C.; Barbu-Tudoran, L.; Pana, O. Enhanced Photocatalytic Activity of Co Doped SnO2 Nanoparticles by Controlling the Oxygen Vacancy States. *Opt. Mater.* **2020**, *110*, 110472. https://doi.org/10.1016/j.optmat.2020.110472.

(13) Al-Baarri, A. N.; Legowo, A. M.; Abduh, S. B. M.; Mawarid, A. A.; Farizha, K. M.; Silvia, M. Production of Ozone and the Simple Detection Using Potassium Iodide Titration Method. *IOP Conf. Ser. Earth Environ. Sci.* **2019**, *292* (1), 012062. https://doi.org/10.1088/1755-1315/292/1/012062.
(14) Thoma, J. A.; French, D. The Starch-Iodine-Iodide Interaction. Part I. Spectrophotometric Investigations1. *J. Am. Chem. Soc.* **1960**, *82* (16), 4144–4147. https://doi.org/10.1021/ja01501a004.
