# Supplementary material for: A Co-Doping Materials Design Strategy for Selective Ozone Electrocatalysts
Source: J Phys Chem Lett. 2024 Jul 11;15(28):7351–6. doi: 10.1021/acs.jpclett.4c01150 (PMC11261613; doi:10.1021/acs.jpclett.4c01150)
Supplement: Supplementary file 2 — jz4c01150_si_002.pdf [file jz4c01150_si_002.pdf]

jz-2024-01150c.R1

Name: Peer Review Information for "A Co-Doping Materials Design Strategy for Selective Ozone Electrocatalysts"

First Round of Reviewer Comments

Reviewer: 1

Comments to the Author

It is a good research work.

Reviewer: 2

Comments to the Author

In this work, the authors propose that selective ozone production of electrocatalysts can be achieved by co-doping tin oxide with two elements, providing a new way of thinking for the advancement of EOP catalyst design. However, there are many concerns that should be addressed. Therefore, the manuscript can be accepted for publication in The Journal of Physical Chemistry Letters after major revision by addressing the following concerns:

1. The authors do not mention in the abstract exactly how much the EOP selectivity of the new catalyst has been improved and whether it has potential for practical application?

2. The test results corresponding to Fig.2 show the oxidation of transition metals and the formation of ·OOH, which does not directly indicate that the co-doping catalysts are favourable for the enhancement of EOP activity. Is it possible to provide more direct evidence that co-doping catalysts are conducive to the enhancement of EOP activity?

3. Considering the completeness of the research work, this work should be supplemented with EOP stability tests of co-doped catalysts under acidic test conditions.

4. "Increased conductivity simultaneously promotes both H_2O_2 generation and its heterogeneous consumption to form O_2, increasing total current but reducing EOP activity and selectivity" mentioned by the authors on page 8, could more direct test evidence be provided to support this conclusion?

5. In Fig.2, it is suggested to use different colored lines to differentiate the test results, which could improve readability.

6. In the sentence “we validate this hypothesis by synthesizing co-doped SnO_2 catalysts with tantalum (Ta), Sb, and tungsten (W) n-type dopants combined with Ni, cobalt (Co), and iron (Fe) as TM dopants” on page 3 of the manuscript, "Sb" should also be written with the full name.

Reviewer: 3

Comments to the Author

1. What is the major advance reported in the paper?

The study investigates tantalum, antimony, and tungsten n-type dopants with nickel, cobalt, and iron as transition metal dopants, and proposes that co-doping tin oxide yields EOP active catalysts. Different from other paper, this work proposes a novel mechanism for the indirect electrosynthesis of ozone, offering significant potential for further exploration of EOP mechanism.

2. What is the immediate significance of this advance?

The study investigate the effect of metal co-doping catalysts on the selectivity of EOP reaction. In addition, hydrogen peroxide (H_2O_2) produced by $2e^-$ water oxidation is catalyzed by leached TM cations to solution-phase hydroperoxyl radicals (•OOH) via a homogenous pseudo-Fenton reaction, these radicals are subsequently electrochemically oxidized to O_3, proposing a novel EOP reaction mechanism. This mechanism can provide relevant guidance and inspiration for the study of ozone electrosynthesis pathway. However, the evidence and data confirming the feasibility of this mechanism are weak, the manuscript needs to be thoroughly reviewed and questioned for further consideration regarding its suitability for the journal.

3. Technical suggestions

Some issues need be resolved.

(1) The characterization peak of Fe can be observed in Figure 1C, contradicting the description provided in lines 54-56 on page 4, which states that only Ni was detected on the film surface, with Co and Fe being undetectable despite their equal molar amounts initially added. It is ambiguous Fe exists due to the coincidence of Fe 2p and Sn 2p3/2 orbitals, and additional characterization is suggested for further confirmation, such as ICP.

(2) The manuscript emphasizes that leached TMs catalyze H_2O_2 to form solution-phase •OOH, which ultimately leads to O_3 production. However, there is no evidence confirming the existence and role of the H_2O_2 intermediate species.

(3) To help the reader understand this sentence please provide relevant literature supporting the statement “Production of •OOH on all nine catalysts is further evidenced by the absorbance spectra of 2-hydroxy ethidium, the selective product of •OOH and dihydroethidium in Figure 2D.”

(4) “W-doped catalysts displayed the highest conductivity, followed by catalysts doped with Sb and Ta.Based on reported variation in literature, we attribute this trend to synthesis conditions rather than intrinsic dopant properties”, Further explanation is needed to clarify whether this trend is attributed to synthesis conditions or intrinsic dopant properties.

(5) “Voltammetry and spectroscopic detection of radicals show that leached TMs catalyze H2O2 to solution phase •OOH”, but, it should be noted that electrochemical experiments alone do not definitively prove the existence of •OOH free radicals.

(6) After leaching of TM cations, whether its catalytic stability will be affected.

(7) Please provide information on the valence band and conduction band of the n-type semiconductor.

(8) How ozone performance is measured.

(9) The manuscript lacks a comparison of transition metals tested individually.

(10) In order to improve the quality of the article, the logic of the manuscript can be further adjusted.

Reviewer: 4

Comments to the Author

This study explored SnO2 doped with n-type dopants (Ta, Sn, W) and transition metal dopants (Ni, Co, Fe) for selective O3 evolution reaction. The behavior of electrodes prepared by modified Pechini method was understood by electrical conductivity and current efficiency. The following comments need to be considered before publication.

1. Fig.2d data should be presented in terms of absorption at 440 nm. Also, control experiments without Ni, Fe, and Co should be performed.

2. The above data can be correlated with the charge efficiency of O3 generation.

3. For fig. 4, detailed experimental conditions should be specified.

4. It’s not fair to compare the efficiency of variable catalysts only at a single potential. The charge efficiency and generation rates of O3 evolution should be compared at several potentials.

5. As the authors noted, there’s no information on the stability. At least, repetitive CVs should be presented to compare stability.

6. The descriptions below eq 2 and 5, ‘6-e- EOP (eq-1) and 4-e- OER (eq-2)’, ‘2-e- H2O2 production (eq-3), pseudo-Fenton •OOH generation (eq-4), and •OOH oxidation to form O3 (eq-5)’ should be removed.

Author's Response to Peer Review Comments:

Thank you for the comments. Our responses are attached.

Dear Reviewers and Editors,

We are grateful for your valuable feedback and the opportunity to revise our manuscript. Your insights have significantly strengthened our work's quality and clarity. We have addressed the reviewers' major revisions by strengthening the evidence for our claims with additional electrochemical stability data, catalyst characterization, and controls. Additionally, we have enhanced clarity and flow throughout the manuscript by refining our discussion and logic, highlighting our focus when necessary, and replotting data for improved readability. Detailed responses to each reviewer's comments are provided below, with our responses in blue text.

Reviewer 1

It is a good research work.

We thank the reviewer for their kind words.

Reviewer 2

In this work, the authors propose that selective ozone production of electrocatalysts can be achieved by co-doping tin oxide with two elements, providing a new way of thinking for the advancement of EOP catalyst design. However, there are many concerns that should be addressed. Therefore, the manuscript can be accepted for publication in The Journal of Physical Chemistry Letters after major revision by addressing the following concerns:

1. The authors do not mention in the abstract exactly how much the EOP selectivity of the new catalyst has been improved and whether it has potential for practical application?

This work presents a new design strategy for EOP-active and selective catalysts. Our main focus on fundamental insight, rather than performance metrics, is now emphasized in the revised manuscript. Notably, the performance of Co and Ni doped catalysts in this study is in range with values reported in literature (refs 4,6,12, and 45), with Ni/Ta-SnO_2 achieving a maximum current efficiency exceeding 60%. However, similar to SnO_2-based catalyst supports for OER and SnO_2-based catalysts for advanced oxidation processes, instability in acid remains a hurdle for practical applications (refs 15,19,46, and 47).

2. The test results corresponding to Fig.2 show the oxidation of transition metals and the formation of ·OOH, which does not directly indicate that the co-doping catalysts are favourable for the enhancement of EOP activity. Is it possible to provide more direct evidence that co-doping catalysts are conducive to the enhancement of EOP activity?

The direct evidence for the success of our proposed co-doping strategy lies in the fact that undoped and singly doped SnO_2 variants (only transition metals or only n-type dopants) do not produce detectable amounts of O_3. Only co-doped SnO_2 produces O_3 where the measured flux and current efficiency of all other materials are zero. This point is now highlighted in the revised manuscript.

3. Considering the completeness of the research work, this work should be supplemented with EOP stability tests of co-doped catalysts under acidic test conditions.

As recommended by the reviewer, the revised SI now contains 24-hour stability tests under potentiostatic conditions (2.70 V vs RHE, 0.5 M H_2SO_4) for all catalysts.

4. "Increased conductivity simultaneously promotes both H_2O_2 generation and its heterogeneous consumption to form O2, increasing total current but reducing EOP activity and selectivity" mentioned by the authors on page 8, could more direct test evidence be provided to support this conclusion?

To fully understand how we deduced that transient H_2O_2 is present in our system, we refer to Ref. 14. Briefly, our previous work on Ni/Sb-SnO_2, which is a well-known catalyst for EOP, suggested that O_3 is generated because leached Ni cations catalyze transient H_2O_2 into homogenous ·OOH radicals which we uniquely linked to the generation of O_3. Based on that work, we have proposed the following mechanism for O_3 generation on SnO_2 -based electrodes:

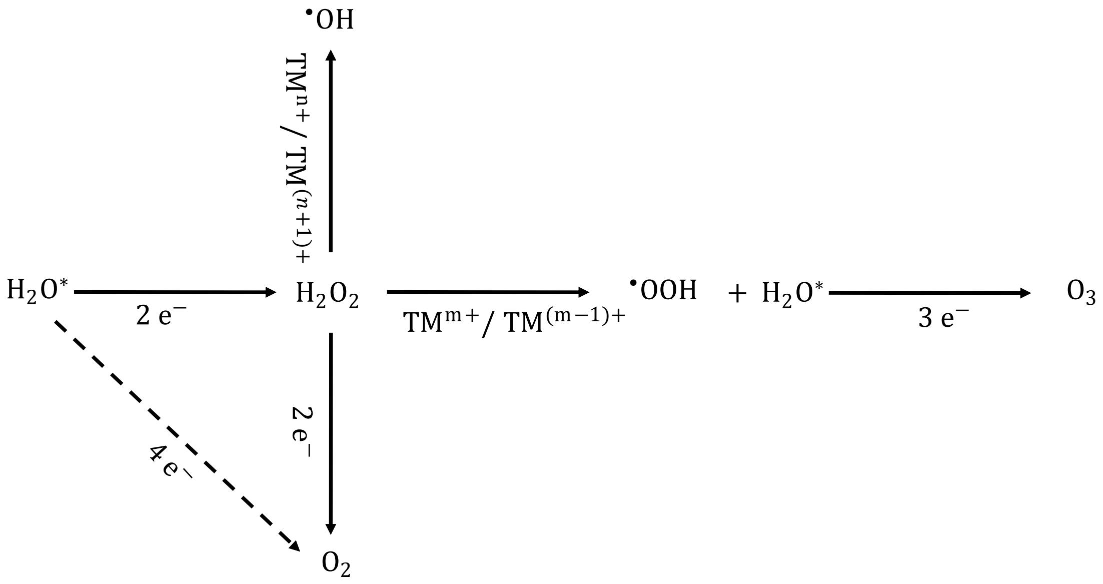

This mechanism was supported by spectroscopic detection of reactive oxygen species (at potentials where they cannot be produced directly from water), electroanalysis, and quantum chemistry calculations.

In this work, we propose that, if this mechanism is correct, any transition metal capable of oxidizing transient H_2O_2 into homogenous ·OOH radicals can replace Ni. Therefore, our goal with Figure 2 was to demonstrate that transition metals are present in multiple oxidation states under reaction conditions and that ·OOH is generated on co-doped SnO_2. To further emphasize this point, we have added control experiments with electrodes lacking TM dopants which did not generate any ·OOH to the SI. This mechanism has also led us to predict that antimony is catalytically inert, and its main function is increasing the catalyst conductivity, and thus can be replaced by other n-type dopants.

As we discussed in Ref. 14, we were unable to detect H_2O_2 directly with chemical titration or other methods. We attribute this inability to rapid conversion from H_2O_2 to radicals (as shown above) as well as electrochemical oxidation to O_2. For reference, the equilibrium potential for $H_2O_2 \rightarrow O_2 + 2H^+ + 2e^-$ is 0.7 V vs RHE, and O_3 is first produced at 2.15 V (ref 14). Thus, while we cannot confirm the presence of H_2O_2 directly, the proposed mechanism explains many experimental observations in our system such as the activity of various Fenton-type dopants and the relationship between conductivity and EOP activity/selectivity.

5. In Fig.2, it is suggested to use different colored lines to differentiate the test results, which could improve readability.

Thank you for this suggestion, which is now implemented in Figure 2.

6. In the sentence “we validate this hypothesis by synthesizing co-doped SnO2 catalysts with tantalum (Ta), Sb, and tungsten (W) n-type dopants combined with Ni, cobalt (Co), and iron (Fe) as TM dopants” on page 3 of the manuscript, "Sb" should also be written with the full name.

Thank you for this suggestion. In response, we have added antimony’s full name to the revised manuscript.

Reviewer: 3

Recommendation: This paper is probably publishable, but major revision is needed; I do not need to see future revisions.

Comments:

1. What is the major advance reported in the paper?

The study investigates tantalum, antimony, and tungsten n-type dopants with nickel, cobalt, and iron as transition metal dopants, and proposes that co-doping tin oxide yields EOP active catalysts. Different from other paper, this work proposes a novel mechanism for the indirect electrosynthesis of ozone, offering significant potential for further exploration of EOP mechanism.

2. What is the immediate significance of this advance?

The study investigates the effect of metal co-doping catalysts on the selectivity of EOP reaction. In addition, hydrogen peroxide (H_2O_2) produced by 2e- water oxidation is catalyzed by leached TM cations to solution-phase hydroperoxyl radicals (˙OOH) via a homogenous pseudo-Fenton reaction, these radicals are subsequently electrochemically oxidized to O3, proposing a novel EOP reaction mechanism. This mechanism can provide relevant guidance and inspiration for the study of ozone electrosynthesis pathway. However, the evidence and data confirming the feasibility of this mechanism are weak, the manuscript needs to be thoroughly reviewed and questioned for further consideration regarding its suitability for the journal.

3. Technical suggestions

Some issues need be resolved.

(1) The characterization peak of Fe can be observed in Figure 1C, contradicting the description provided in lines 54-56 on page 4, which states that only Ni was detected on the film surface, with Co and Fe being undetectable despite their equal molar amounts initially added. It is ambiguous Fe exists due to the

coincidence of Fe 2p and Sn 2p3/2 orbitals, and additional characterization is suggested for further confirmation, such as ICP.

We appreciate the recommendation provided by the reviewer. The peak present near 716 eV corresponds to Sn $2p_{3/2}$ and not Fe 2p (ref 26), which would appear as doublets (one peak for Fe $2p_{3/2}$ and one for Fe $2p_{1/2}$). Therefore, the detection of any Fe present on the film surface with XPS would be obscured by tin due to the low doping ratio. We have revised the manuscript and remade Figure 1C to elaborate on this ambiguity.

Furthermore, we have included CVs for Fe-SnO_2 prepared using the conventional method and the modified Pechini method to the SI. These CVs reveal the absence of oxidation peaks associated with Fe when using the conventional method, thereby supporting our assertion that a larger amount of Fe is incorporated with the modified Pechini method.

(2) The manuscript emphasizes that leached TMs catalyze H2O2 to form solution-phase ˙OOH, which ultimately leads to O3 production. However, there is no evidence confirming the existence and role of the H_2O_2 intermediate species.

Please see our response to Reviewer 2, #4.

(3) To help the reader understand this sentence please provide relevant literature supporting the statement "Production of ˙OOH on all nine catalysts is further evidenced by the absorbance spectra of 2-hydroxy ethidium, the selective product of •OOH and dihydroethidium in Figure 2D."

Thank you for this suggestion. In response, we have added references (30-32) to support our claims.

(4) "W-doped catalysts displayed the highest conductivity, followed by catalysts doped with Sb and Ta. Based on reported variation in literature, we attribute this trend to synthesis conditions rather than intrinsic dopant properties," Further explanation is needed to clarify whether this trend is attributed to synthesis conditions or intrinsic dopant properties.

Thank you for this suggestion. We have rephrased this part and added references (18,42-44) to emphasize that we do not claim universal relationships between dopant identity and SnO_2 conductivity across varying synthesis conditions and dopant ratios.

(5) “Voltammetry and spectroscopic detection of radicals show that leached TMs catalyze H2O2 to solution phase •OOH”, but, it should be noted that electrochemical experiments alone do not definitively prove the existence of •OOH free radicals.

As recommended by the reviewer, we have revised the statement to acknowledge the limitations in our findings.

(6) After leaching of TM cations, whether its catalytic stability will be affected.

TM cation leaching does not directly affect catalytic stability, as discussed in Ref 14.

(7) Please provide information on the valence band and conduction band of the n-type semiconductor.

Thank you for this suggestion. In response, we have added additional characterization of un-doped SnO_2 which includes Tauc plot, CV, and XRD to the SI. Additionally, we have elaborated the explanation of the rationale behind n-type doping SnO_2 in the main manuscript.

(8) How ozone performance is measured.

Dissolved O_3 concentration is measured using its direct UV absorbance at 258 nm. This method is detailed extensively in the experimental section of our SI, and it is commonly used among researchers in the field.

(9) The manuscript lacks a comparison of transition metals tested individually.

Singly TM-doped SnO_2, devoid of n-type dopants, do not generate detectable O_3 which we attribute to their low electrical conductivity, as shown in Figures 3 and 4, resulting in diminished electrochemical performance. To substantiate this claim, we have included control CVs in the SI, demonstrating the low electrochemical performance of these electrodes.

(10) In order to improve the quality of the article, the logic of the manuscript can be further adjusted.

To enhance the overall quality of the article, we have refined the logical flow of the manuscript by rephrasing certain sections, remaking plots, and further explaining our co-doping strategy.

Reviewer: 4

Recommendation: This paper is probably publishable, but major revision is needed; I do not need to see future revisions.

Comments:

This study explored SnO2 doped with n-type dopants (Ta, Sn, W) and transition metal dopants (Ni, Co, Fe) for selective O3 evolution reaction. The behavior of electrodes prepared by modified Pechini method was understood by electrical conductivity and current efficiency. The following comments need to be considered before publication.

1. Fig.2d data should be presented in terms of absorption at 440 nm. Also, control experiments without Ni, Fe, and Co should be performed.

Thank you for your suggestion. Figures S10 shows control experiments with singly n-type doped SnO_2 without transition metal dopants. The plots were also adjusted to display absorption values.

2. The above data can be correlated with the charge efficiency of O3 generation.

Correlating the concentration of ·OOH with the concentration of O_3 is a great suggestion worthy of consideration for future investigations. However, it is essential to note the probe utilized in this study is primarily suited for qualitative assessments. While dihydroethidium selectivity reacts with ·OOH to generate 2-hydroxyethdium, it can also undergo a sluggish non-selective oxidation process, resulting in the formation of ethidium, which overlaps with the absorbance spectrum of 2-hydroxyethdium (ref 30-32). Moreover, inconsistencies in the available data regarding the extinction coefficient in different electrolytes further compound the challenge of achieving precise quantitative measurements.

3. For fig. 4, detailed experimental conditions should be specified.

Thank you for this suggestion. We have included a brief description delineating the experimental conditions pertaining to the test depicted in Figure 4 within the caption. Comprehensive details regarding the experimental setup can be found in the Methods section of the SI.

4. It's not fair to compare the efficiency of variable catalysts only at a single potential. The charge efficiency and generation rates of O_3 evolution should be compared at several potentials.

We acknowledge the reviewer's valid point regarding the comparison of catalyst performance at a single potential, which does not provide a comprehensive assessment of their relative activity/selectivity. However, our goal is not to determine the superiority of specific dopants for EOP. We rather seek to illustrate the effectiveness of our proposed co-doping strategy for EOP-active and selective catalysts. Additionally, we aim to explain variations in performance in the context of the proposed mechanism. We have revised the manuscript to emphasize our focus.

5. As the authors noted, there's no information on the stability. At least, repetitive CVs should be presented to compare stability.

Thank you for this suggestion. 24-hour stability tests have now been added to the SI.

6. The descriptions below eq 2 and 5, '6-e- EOP (eq-1) and 4-e- OER (eq-2)', '2-e- H_2O_2 production (eq-3), pseudo-Fenton •OOH generation (eq-4), and •OOH oxidation to form O_3 (eq-5)' should be removed.

Thank you for this suggestion. In response, we have removed these descriptions.
